# Connexin 43 mediated gap junctional communication enhances breast tumor cell diapedesis in culture

**DOI:** 10.1186/bcr1042

**Published:** 2005-05-13

**Authors:** Mary-Ann Pollmann, Qing Shao, Dale W Laird, Martin Sandig

**Affiliations:** 1 Department of Anatomy and Cell Biology, The University of Western Ontario, London, Ontario, Canada

## Abstract

**Introduction:**

Metastasis involves the emigration of tumor cells through the vascular endothelium, a process also known as diapedesis. The molecular mechanisms regulating tumor cell diapedesis are poorly understood, but may involve heterocellular gap junctional intercellular communication (GJIC) between tumor cells and endothelial cells.

**Method:**

To test this hypothesis we expressed connexin 43 (Cx43) in GJIC-deficient mammary epithelial tumor cells (HBL100) and examined their ability to form gap junctions, establish heterocellular GJIC and migrate through monolayers of human microvascular endothelial cells (HMVEC) grown on matrigel-coated coverslips.

**Results:**

HBL100 cells expressing Cx43 formed functional heterocellular gap junctions with HMVEC monolayers within 30 minutes. In addition, immunocytochemistry revealed Cx43 localized to contact sites between Cx43 expressing tumor cells and endothelial cells. Quantitative analysis of diapedesis revealed a two-fold increase in diapedesis of Cx43 expressing cells compared to empty vector control cells. The expression of a functionally inactive Cx43 chimeric protein in HBL100 cells failed to increase migration efficiency, suggesting that the observed up-regulation of diapedesis in Cx43 expressing cells required heterocellular GJIC. This finding is further supported by the observation that blocking homocellular and heterocellular GJIC with carbenoxolone in co-cultures also reduced diapedesis of Cx43 expressing HBL100 tumor cells.

**Conclusion:**

Collectively, our results suggest that heterocellular GJIC between breast tumor cells and endothelial cells may be an important regulatory step during metastasis.

## Introduction

Tumor metastasis is a multi-step process that involves the dissociation of tumor cells from the primary tumor followed by their entry into the circulation, extravasation from the vascular system and proliferation into tumor masses at secondary tissue sites. Both cell-cell and cell-matrix interactions are important regulators at different stages of this metastatic cascade [1-4]. For instance, loss of E-cadherin in bladder, prostate, breast and colorectal cancers, or integrins such as α2β1 and α5β1 in breast cancer and α6β1 in melanoma cells correlates with increased malignancy of tumor cells, indicating that the integrity of the primary tumor depends on signals from adjacent cells and from appropriate extracellular matrix ligands [1,2,5-8].

Extravasation of malignant cells often involves transendothelial migration (diapedesis) into tissues prior to forming secondary tumors. In contrast to diapedesis of leukocytes during inflammatory responses, little is known about the molecular mechanisms that regulate tumor cell diapedesis. The integrity of the endothelium depends mainly on the organization of interendothelial junctions, and tumor cells must traverse these junctions to extravasate across the endothelial barrier. Our previous studies demonstrated that diapedesis of melanoma cells is in part regulated by adhesion receptors present on both the vascular endothelium and the tumor cell [9]. A localized disruption in the endothelium of vascular endothelial (VE)-cadherin, α-catenin and platelet endothelial cell adhesion molecule-1 (PECAM-1) occurs at the site of tumor cell penetration, which is restored once the tumor cell completes diapedesis [9]. We further demonstrated that endothelial N-cadherin is necessary for the completion of melanoma cell diapedesis, suggesting that endothelial cells actively participate in the process of tumor cell diapedesis [9]. Integrins appear to play an important role during extravasation of tumor cells by binding to components in the extracellular matrix and on endothelial cells. The integrin αvβ3 regulates diapedesis of melanoma cells by binding to the immunoglobulin like adhesion receptor L1 on endothelial cells [10]. In prostate tumor cells, β3 integrins regulate diapedesis by binding to matrix components underneath the endothelium [11] and, in breast tumor cells, integrins β1, α5 and αvβ3 are involved in adhesion and migration through the extracellular matrix components vitronectin and fibronectin [12]. In addition, it has been reported that integrins mediate invasion and metastasis in murine melanoma cells and gliomas [13,14].

These previous studies suggest that signaling via cell-cell and cell-matrix adhesion receptors facilitates tumor cell diapedesis. Another possible mechanism by which tumor cells may communicate with endothelial cells to cross the endothelial barrier involves gap junctions [15-17]. Gap junctions are intercellular channels that mediate the direct intercellular exchange of secondary messengers, small metabolites or inorganic ions [18]. These channels are located at the plasma membrane and are composed of two hemichannels called connexons, each of which is assembled from six oligomerized protein subunits called connexins (Cx) [18,19]. To date the connexin family includes 20 members named according to their predicted molecular weights [20]. Connexin expression varies between cell types, and individual cells often express more than one connexin family member [18,21]. Although endothelial cells are heterogeneous according to the size of the vessel or the vascular bed of origin, they all express three different types of connexin, Cx43, Cx37 and Cx40 [22-26]. In normal human mammary glandular epithelium, Cx43 is found most often between mammary myoepithelial cells and in luminal cells, while adjacent luminal cells predominantly assemble Cx26 gap junctions [27,28]. Studies have demonstrated a decrease in gap junctional intercellular communication (GJIC) and connexin expression or aberrant connexin trafficking and assembly in primary mammary tumors [29-31]. Little is known, however, about connexin expression and GJIC in tumor cells *en route *to secondary metastatic sites outside the site of the primary diseased tissue. Previous studies have observed apparent gap junction formation between mammary tumor and vascular endothelial cells but the consequences of this interaction were not established [15,16]. In another study, Cx26 expressing BL6 mouse melanoma cells were found to form tumor/endothelial cell heterocellular gap junctions and these connexin expressing tumor cells had increased metastatic properties *in vivo *[17]. These findings are puzzling as Cx26 is not generally capable of forming heterotypic gap junctions with any of the endothelial expressed connexins, which include Cx40, Cx37 and Cx43 [32].

Previous studies investigating GJIC between tumor cells and endothelial cells focused primarily on tumor cell adhesion to the endothelium during the initial stages of diapedesis [15-17]. To address the hypothesis that GJIC may affect diapedesis, we used a non-metastatic cell line (HBL100) that is GJIC-deficient and does not express any known connexins in order to better evaluate a possible contribution of GIJC to diapedesis. Because endothelial cells abundantly express Cx43, we engineered HBL100 cells to express either wild-type Cx43 or a non-functional chimeric mutant of Cx43 to determine if the potential of HBL100 cells to cross the endothelium would change under both or either of these conditions. In this report we show that Cx43 expression up-regulates tumor cell diapedesis via a GJIC-dependent mechanism.

## Materials and methods

### Cell lines and culture conditions

Human microvascular endothelial cells (HMVECs) derived from the lung were purchased from Cambrex Biosciences Inc. (Walkersville, MD, USA) and cultured according to supplier's instructions in endothelial growth medium (EGM; Cambrex Biosciences Inc.), supplemented with 100 units/ml penicillin G sodium, and 100 μg/ml streptomycin sulphate (Invitrogen, Burlington, ON, Canada). Wild-type transformed mammary epithelial cells (HBL100) and HBL100 cells expressing Cx43 or Cx43 with green fluorescent protein (GFP) tagged to the amino terminal (GFP-Cx43) [33] were routinely cultured in D-MEM supplemented with 100 units/ml penicillin G sodium, 100 μg/ml streptomycin, 2 mM L-glutamine (Invitrogen) and 10% FBS (Sigma-Aldrich, Oakville, ON, Canada). All cells were maintained in a humidified chamber at 37°C containing 5% CO_2_.

### Constructs, infection and transfection

HBL100 cells expressing Cx43 (HBL100Cx43) or empty vector (HBL100v) were engineered by retroviral infection as described by Qin *et al*. [34]. Briefly the cDNA of Cx43 inserted into the AP-2 retroviral vector was transfected into the 293GPG packaging cell line and 48 h later retroviral supernatant was collected and filtered through a 0.45 μm filter and used to infect HBL100 cells. Twenty-four hours after infection, medium was replaced with D-MEM and cells were subcultured as normal. More than 90% of tumor cells expressed Cx43 after three rounds of viral infection as determined by immunofluorescence [34].

HBL100 cells expressing non-functional GFP-Cx43 (HBL100GFP-Cx43) [33] were generated by transfecting 2.5 × 10^5 ^HBL100 cells with 2 μg of GFP-Cx43 using Metafectene (Biontex Laboratories GmbH, Munich, Germany). After 24 h, approximately 40% of cells expressed GFP-Cx43 and these cells were used for diapedesis assays.

### Diapedesis assay

The diapedesis assay was carried out as previously described [9]. Briefly, 7.5 × 10^5 ^HMVECs were seeded onto 12 mm glass coverslips coated with matrigel (Becton-Dickenson, Bedford, MA, USA). HMVEC cells were allowed to settle for 2–3 h and form monolayers in a 37°C humidified chamber containing 5% CO_2_. Coverslips were then transferred to a 24-well plate and cultured for 48 h in EGM. The number of cells seeded produces an endothelial monolayer without requiring cell proliferation. Monolayers have intact adherens junctions as previously evaluated by VE-cadherin staining [9,35]. HBL100 tumor cells were labeled with 10 μg/ml 1,1'-dioctadecyl-3, 3,3', 3'-tetramethylindocarbocyanine percholate (DiI; Molecular Probes, Eugene, OR, USA), and harvested non-enzymatically using 2 mM EDTA (EMD Chemicals Inc., Gibbstown, NJ, USA). Approximately 7.5 × 10^4 ^cells were added to endothelial monolayers at a ratio of around 1:10 (tumor cell:endothelial cell). Cells were then co-cultured for different times (1, 5, or 7 h) before live observation or fixation and immunostaining. Diapedesis was quantified blinded to the investigators using a Leica IRE2-DM epifluorescence microscope (Leica Microsystems Inc., Toronto, ON, Canada) as previously described [11]. Briefly, co-cultures were fixed and stained for F-actin. Diapedesis was quantified by counting the number of tumor cells in contact with the endothelium classified into three stages according to their position relative to the endothelium: round on top (tumor cells with a spherical shape located on the apical surface of the endothelium); migrating (tumor cells have penetrated through the endothelial monolayer at endothelial cell junctions where part of the cell body is located above the endothelium and part of it has spread on the matrigel, beneath the endothelial cell f-actin stress fibers); and underneath (all of the tumor cell body is below the plane of the endothelial cell stress fibers). Migrating and underneath cells were scored together as transmigrating. For each coverslip, approximately 100 cells adherent to the endothelium were scored and counted and all experiments were repeated three times with triplicate coverslips.

### Pre-loading dye coupling assay

GJIC was evaluated using the preloading dye coupling assay as described previously [36] with the following modifications. Tumor cells were labeled for 15 minutes at 37°C with 2 ml of Opti-MEM (Invitrogen) containing 10 μg/ml calcein-AM and 10 μg/ml DiI. Calcein-AM is a fluorescent substrate that is cleaved in viable cells into a membrane impermeable form able to pass through functional gap junctions but not through other plasma membrane channels. Labeled tumor cells were washed twice with Hank's balanced salt solution (HBSS), harvested non-enzymatically using 2 mM EDTA, and added to HMVEC monolayers on matrigel. Dye transfer from tumor cells to endothelial cells was observed live by epifluorescence microscopy after 30 minutes of co-culture and the number of adherent tumor cells that transferred dye to adjacent endothelial cells was scored and expressed as percent of total number of tumor cells counted. For each coverslip, approximately 70 cells adherent to the endothelium were scored. Unless specified, all experiments were repeated three times with triplicate coverslips.

### GJIC as assessed by fluorescence recovery after photobleaching

To determine whether carbenoxolone (CBX) blocks gap junctional coupling, HMVEC monolayers were pretreated for 7 h with 150 μM CBX or the inactive analog glycyrrhizic acid (GZA). HMVEC were then loaded for 15 minutes with 10 μg/ml calcein-AM (Molecular Probes) in an Opti-MEM solution containing CBX or GZA. Cells were rinsed twice with Opti-MEM containing CBX or GZA and immersed in fresh media containing CBX or GZA. Because the fluorescent dye can only pass through gap junctions, recovery of fluorescence in the bleached cell will only occur if dye passes through gap junctions with adjoining unbleached cells. For each experimental condition, three individual cells were bleached for approximately 30 s using the Zeiss laser scanning confocal microscope (LSM) 410 and an argon laser at full power. To observe recovery of fluorescence, images were obtained every five minutes following photobleaching using the argon laser at 33% power until maximum recovery was reached (15 minutes). In control experiments, monolayers were left untreated or were treated with the inactive analog of CBX, GZA. Mean fluorescence intensity of bleached/unbleached cells in the same area was measured over time using scion imaging software and expressed as mean relative recovery (arbitrary units). Data represent three independent experiments.

### Immunofluorescence staining and laser scanning confocal microscopy

Cells were fixed at room temperature for 20 minutes with 2% or 4% (w/v) paraformaldehyde (EMD Chemicals Inc.) and washed in cation free phosphate buffered saline (PBS). Cells were permeabilized for five minutes at 4°C using a buffer containing 15 mM Tris-HCL, 120 mM sodium chloride (NaCl), 25 mM potassium chloride (KCl), 2 mM EDTA, 2 mM EGTA, 0.5% (v/v) Triton X-100, pH 7.5. To label F-actin, co-cultures were incubated for 30 minutes at room temperature with Alexa Fluor^® ^488 or Texas Red conjugated-phalloidin (Molecular Probes) diluted 1:50 in PBS with 1% (w/v) bovine serum albumin (BSA) followed by three washes in PBS. To examine the localization of F-actin and Cx43, cells were labeled with Texas red conjugated-phalloidin (1:50 dilution) and with monoclonal anti-Cx43 (clone P4G9 from the Fred Hutchinson Cancer Research Center Antibody Development Group, Seattle, WA, USA) diluted 1:10 in 1% (w/v) BSA/PBS. Cells were incubated with Alexa Fluor^® ^488-conjugated goat-anti-mouse secondary antibody (Molecular Probes). Nuclei were stained using 10 μg/ml Hoechst 33342 (Sigma-Aldrich) diluted in PBS. Coverslips were mounted on glass slides using vectashield mounting media (Vector Laboratories, Burlington, ON, Canada) and sealed with nail enamel. The inclusion of spacers made from plastic coverslips cut into strips prevented contact of co-cultures with the slides as described by Voura *et al*. [37].

Co-cultures were imaged using a Zeiss LSM 410 laser scanning confocal microscope system (Carl Zeiss Canada Ltd, Toronto, ON, Canada). Serial 0.7 μm optical sections of selected areas were recorded and evaluated with the LSM 410 software (Carl Zeiss Canada Ltd). Representative serial optical sections are presented in an apical to basal direction.

### Adhesion assay

Adhesion to the endothelium was evaluated as previously described for leukocyte adhesion to the endothelium [38] with the following modifications. HMVEC monolayers were established in 1:20 matrigel coated wells (2.1 × 10^4 ^cells per well) and cultured for 48 h. HBL100, HBL100v, or HBL100Cx43 cells (2.1 × 10^3^), pre-labeled with DiI, were added to HMVEC monolayers and co-cultured for 1 h. Co-cultures were fixed with 2% (w/v) paraformaldehyde and washed twice with PBS to remove non-adherent cells. The PBS was replaced with water and fluorescence intensity of DiI was measured for each well at 549 nm excitation and 565 nm emission wavelengths using a SAFIRE fluorescent microplate reader (Tecan US Inc., Durham, NC, USA). The number of adherent tumor cells was calculated and normalized based on a standard curve generated with known numbers of DiI-labeled tumor cells present in duplicate wells in the same microplate. Experiments were repeated three times with eight sample replicates per cell type.

### Protein extraction and western blot analysis

HBL100, HBL100v, HBL100Cx43, HBL100GFP-Cx43, and HMVEC cells grown in culture dishes were lysed using the following buffer for protein collection: 10 mM Tris (pH 7.4), 150 mM NaCl, 1 mM EDTA, 0.1% (w/v) SDS, and 0.5% (v/v) Triton X-100. Protein concentration was determined with bicinchoninic acid (BCA) protein assay reagent (Pierce Chemical Co., Rockford, IL, USA). Protein (50 μg) for each cell type was separated in 12% SDS-polyacrylamide gels and transferred to nitrocellulose membranes. Nitrocellulose membranes were blocked with 5% non-fat dry milk in tris buffered saline (TBS) containing 5% Tween 20. Membranes were then probed for Cx43 protein, using a monoclonal anti-Cx43 antibody against amino-terminal amino acid residues 1–20 (clone P1E11 from the Fred Hutchinson Cancer Research Center Antibody Development Group, Seattle, WA, USA) at a dilution of 1:500. Immunoblots were probed with appropriate secondary antibodies conjugated to horse radish peroxidase (Pierce Chemical Co.) at a dilution of 1:10,000. The protein was visualized using Supersignal West Pico Chemiluminescent substrate (Pierce Chemical Co.) by exposing the immunoblot to Amersham Hyperfilm (Amersham Biosciences, Piscataway, NJ, USA) for 30 s.

### Statistical analysis

Comparisons between cell types and different time points or treatments were evaluated using a two-way analysis of variance followed by Bonferroni post-hoc test. A p-value of < 0.05 was considered to be significant. Comparisons between cell types at a single time point were evaluated using one-way analysis of variance followed by Tukey's multiple comparison post-hoc test. Data are presented as means +/- SEM.

## Results

### Morphological evaluation of tumor cell diapedesis

**Figure 1:**
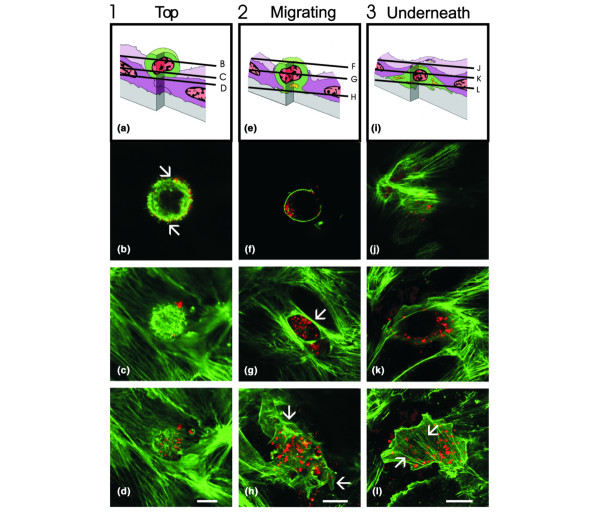
Tumor cell diapedesis through human microvascular endothelial cell (HMVEC) monolayers. HBL100 cells were pre-labeled with dioctadecyl-3, 3,3', 3'-tetramethylindocarbocyanine percholate (DiI; red), co-cultured with HMVEC monolayers for various time periods, fixed with paraformaldehyde and labeled for F-actin (green). Optical sections obtained by laser scanning confocal microscopy at the focal levels indicated in **(a,e,i) **identified three major stages of diapedesis: round on top of **(a-d)**, migrating through **(e-h)**, or located underneath **(i-l) **the endothelium. **(b) **Cells round on top had filopodial extensions present on the apical surface of a cell (arrows). **(g) **Thick bundles of F-actin were present at the interface between endothelial cells and the migrating tumor cell (arrow). **(h) **Migrating tumor cells with portions spread underneath the endothelium (arrows) and **(i) **those that completed diapedesis often contained prominent stress fibers underneath the endothelium (arrows). No major morphological differences between wild-type and connexin43 (Cx43) expressing HBL100 cells were observed. Bar = 10 μm.

Previous work suggested that GJIC between tumor cells and endothelial cells may affect diapedesis during metastasis [15-17]. To analyze and evaluate the efficiency of breast tumor cell diapedesis we used an *in vitro *assay in which tumor cells were allowed to migrate through a monolayer of endothelial cells grown on matrigel as we have previously described [9,39-41]. This assay mimics aspects of the vessel wall and has been used by us [35,42] and others [39-41] to analyze leukocyte diapedesis. Diapedesis was assessed by evaluating the spatial location of tumor cells as they migrated through an endothelial monolayer. Tumor cells pre-labeled with DiI were seeded for 1, 5 or 7 h onto endothelial monolayers before fixation and stained for F-actin. Tumor cells were scored according to their morphology and location with respect to the endothelium. We defined three distinct stages of migration observable by confocal microscopy (Fig. 1): top; migrating; and underneath. Tumor cells located on top of the endothelium prior to diapedesis tended to have a round or oval shape with small filopodia extending from the periphery of the cell (Fig. 1b, arrows). This shape was maintained from the apical (Fig. 1b) to the basal surface (Fig. 1d) of the tumor cell, differing from melanoma cells, which tend to have a fibroblastic shape and spread on the apical endothelial surface [37]. At sites of tumor cell-endothelial cell interactions, prominent microfilament bundles within the underlying endothelial cell were seen, indicating no disruption of the endothelium (Fig. 1d). Tumor cells in the process of diapedesis (migrating) had a unique morphology with distinct shapes at various focal levels (Fig. 1f–h) not previously seen in melanoma cells [37] but very similar to transmigrating monocytes and prostate tumor cells [11,35]. The apical aspect of migrating tumor cells located above the endothelium was round in shape and lacking filopodial extensions, but contained smooth cortical actin staining (Fig. 1f). At the level of the endothelium, tumor cells maintained close contact with the endothelial cells, which formed a circular opening surrounded by thick bundles of F-actin (Fig. 1g, arrow). The basal aspect of the tumor cells was located underneath the endothelium and spread along the matrigel by extending processes in various directions (Fig. 1h, arrows). Tumor cells located completely underneath the endothelium (Fig. 1j–l) had endothelial microfilament bundles extended over the top of the tumor cell as previously seen in melanoma cells [37]. No opening above the tumor cell remained, indicating complete closure of the transmigration passage (Fig. 1j). Tumor cells underneath the endothelium spreading on the surface of the matrigel had an irregular shape and stress fibers (Fig. 1l, arrows). The morphological features of HBL100v or HBL100Cx43 cells (not shown) during diapedesis were not noticeably different from those of HBL100 wild-type cells. We used the morphological criteria shown in Fig. 1 to quantify various stages and assess the efficiency of diapedesis.

### Expression and distribution of exogenous Cx43 in HBL100 cells and HMVEC monolayers

**Figure 2:**
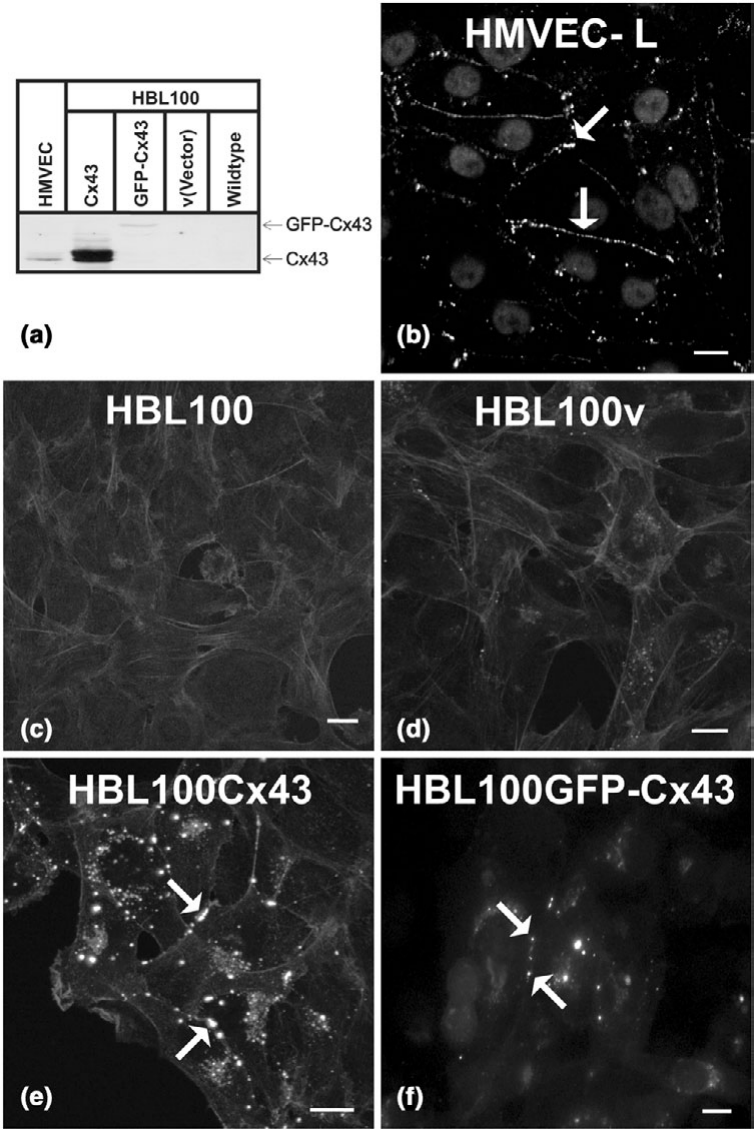
Exogenous connexin43 (Cx43) in HBL100 cells forms gap junctional plaques. To explore if Cx43 expression and/or gap junctional intercellular communication (GJIC) affects diapedesis of breast tumor cells we used GJIC-deficient HBL100 cells to engineer cells expressing functional Cx43 (HBL100Cx43) and non-functional Cx43 (HBL100GFP-Cx43). HBL100v, containing the empty expression vector, served as a control. **(a) **Cx43 expression of these cells was compared by western blot analysis to that of human microvascular endothelial cell (HMVEC) grown on matrigel used as a control for endogenous Cx43 expression. Whereas Cx43 was undetectable in HBL100 and HBL100v cells, HMVEC grown on matrigel, HBL100Cx43, and HBL100GFP-Cx43 cells expressed Cx43. Immunocytochemistry localized Cx43 at cell-cell borders in **(b) **HMVEC monolayers (arrows), **(e) **between HBL100Cx43 cells (arrow) or **(f) **HBL100GFP-Cx43 cells (arrows), whereas **(c) **HBL100 and **(d) **HBL100v cells lacked Cx43 staining. Bar = 10 μm.

To explore if Cx43 expression in HBL100 cells affects any aspects of diapedesis, cells were engineered to express functional Cx43, non-functional GFP-Cx43 or the empty retroviral vector as a control. Cx43 was absent in both wild-type and empty vector HBL100v control cell lines used (Fig. 2a,c,d) but expressed in HMVEC (Fig. 2a,b) and cells engineered to express Cx43 (Fig. 2a,e) or GFP-Cx43 (Fig. 2a,f). Importantly, both Cx43 and GFP-Cx43 were routinely observed to localize to the cell surface, consistent with the formation of gap junction plaques (Fig. 2b,e,f). Intracellular populations of Cx43 likely reflecting different stages of assembly or degradation in HMVEC, HBL100Cx43 and HBLGFP-Cx43 were also seen. These results suggest that exogenous or endogenously expressed Cx43 assembles into gap junctional plaques at sites of homocellular endothelial or tumor cell-cell contacts.

### HBL100vCx43 cells form functional gap junctional channels with HMVEC monolayers

**Figure 3:**
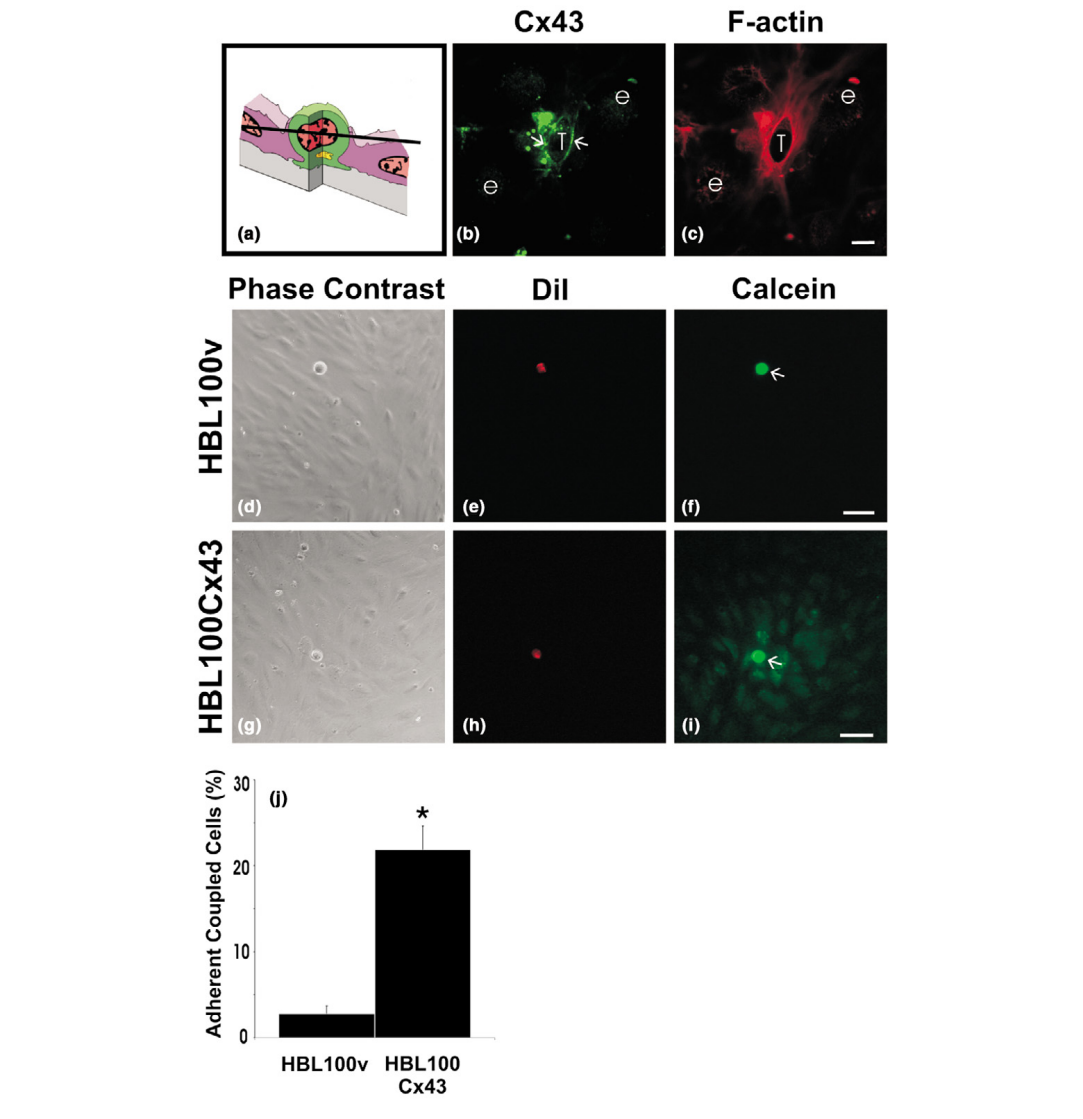
Connexin43 (Cx43) localizes to tumor/endothelial cell interfaces and assembles into functional gap junction channels. To examine Cx43 distribution during tumor cell diapedesis, cells expressing functional Cx43 (HBL100Cx43) were co-cultured with human microvascular endothelial cell (HMVEC) monolayers, fixed and labeled for **(b) **Cx43 (green) and **(c) **F-actin (red). The optical section obtained by confocal microscopy revealed **(b) **Cx43 (arrows) enriched at tumor cell (T)/endothelial cell (e) contact site, where it partially co-localizes with cortical F-actin. **(a) **Schematic diagram indicating the level of the optical section where the tumor cell is wedged between the endothelial cells and **(b) **the location of the Cx43-containing plaques (arrows). To evaluate if expression of exogenous Cx43 in HBL100 cells resulted in formation of functional gap junctions with HMVEC, **(f) **HBL100v or **(i) **HBL100Cx43 cells (arrows) were preloaded with **(e,h) **dioctadecyl-3, 3,3', 3'-tetramethylindocarbocyanine percholate (DiI; red) and **(f,i) **calcein-AM (green) and co-cultured with HMVEC monolayers for 30 minutes. Live observation of the co-cultures revealed that in contrast to **(d-f) **HBL100v cells, **(g-i) **HBL100Cx43 cells allowed the calcein dye to spread to adjacent HMVEC cells. **(j) **Gap junctional intercellular communication (GJIC) between tumor cells and endothelial cells was quantified live after 30 minutes of co-culture, revealing a 10-fold increase in the number of connexin expressing tumor cells coupled to the endothelium compared to tumor cells lacking Cx43 (*p < 0.05). Bar = **(c) **10 μm and **(f,i) **50 μm.

To determine whether HBL100 cells engineered to exogenously express Cx43 formed gap junctions and established GJIC with HMVEC cells, we co-cultured HBL100Cx43 with HMVEC monolayers, localized Cx43 in these cultures (Fig. 3a–c), and assayed for dye transfer between tumor and endothelial cells (Fig. 3d–j). Confocal microscopy revealed Cx43 at tumor cell-endothelial cell interfaces (Fig. 3b, arrows) where tumor cells were wedged between endothelial cells and where cortical F-actin was found (Fig. 3b,c). These results indicate that during diapedesis HBL100Cx43 cells form heterocellular gap junctions with adjacent endothelial cells.

Preloading dye transfer studies to assess whether Cx43 is being assembled into functional gap junctions at tumor/endothelial cell interfaces revealed that dye spread extensively from Cx43 expressing HBL100 cells to endothelial cells (Fig. 3i), whereas dye failed to spread from control HBL100v cells to endothelial cells (Fig. 3f). These results suggest that exogenous Cx43 in HBL100Cx43 can form functional gap junctions with HMVEC early on during diapedesis. Live quantification of coupling performed after 30 minutes of co-culture revealed that ten times more Cx43 expressing HBL100 cells coupled to the endothelium than those lacking Cx43 (Fig. 3j). The low occurrence of dye transfer between HBL100v control cells and HMVEC is likely due to nonspecific dye uptake from damaged tumor cells rather than functional gap junctional coupling due to connexin expression.

### Cx43 enhances HBL100 diapedesis

**Figure 4:**
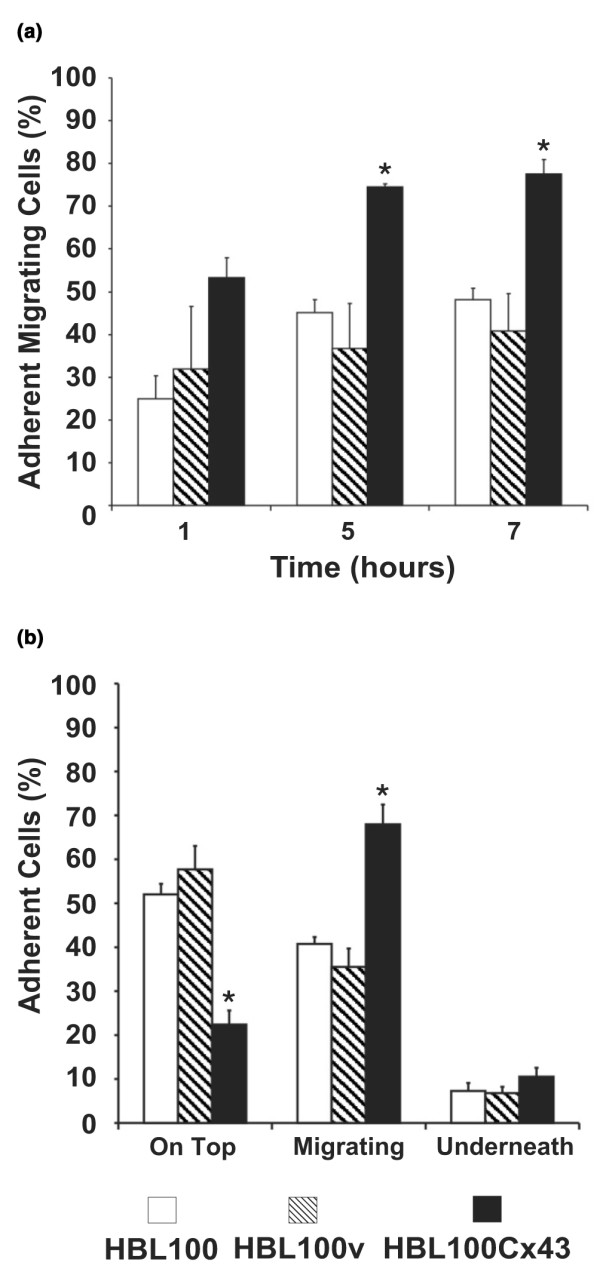
Connexin43 (Cx43) enhances tumor cell diapedesis. HBL100 and HBL100v cells or cells expressing functional Cx43 (HBL100Cx43) were co-cultured with HMVEC monolayers for 1, 5 and 7 h. **(a) **Adherent cells in the process of diapedesis and cells that had completed transmigration were scored together as migrating. Compared to HBL100v or HBL100 wild-type cells a two-fold increase in diapedesis of HBL100Cx43 cells was seen at 1, 5 and 7 h. **(b) **At 7 h the relative proportion of cells at different stages of migration was evaluated. Compared to control cells, the observed two-fold increase in the number of transmigrating HBL100Cx43 cells correlated with a two-fold decrease in the number of HBL100Cx43 cells located on top of the monolayer. No significant difference in the proportion of cells underneath the endothelium was observed between the Cx43 expressing and non-expressing HBL100 cells. Data are expressed as the mean percentage ± SEM of three independent experiments performed in triplicate (*p < 0.05).

To compare the efficiency of diapedesis of Cx43 deficient tumor cells with those that exogenously express Cx43, we co-cultured HBL100, HBL100v or HBL100Cx43 cells with HMVEC for 1, 5 and 7 h. At all time points HBL100Cx43 cells showed an up to two-fold increase in diapedesis efficiency compared to cells lacking Cx43 (Fig. 4a). This increase in migration appeared to plateau at 5 h. Conversely, at all time points no statistical difference in migration efficiency was observed between wild-type (HBL100) and control (HBL100v) cells (Fig. 4a). The relative proportion of cells at different stages of migration was evaluated after 7 h of co-culture (Fig. 4b). About 50% of wild-type and vector control cells encountered were located on top of the endothelium, 40% were migrating across the endothelium, and about 10% were completely located underneath. In contrast, only 20% of HBL100Cx43 cells were located on top of the endothelium, while over 60% migrated across. No significant difference in the relative proportion of cells underneath the endothelium was observed between HBL100 cells with or without Cx43 expression (Fig. 4b). These results suggested that Cx43 facilitated tumor cell diapedesis but it was unclear if this was due to increased adhesiveness or whether GJIC was required.

**Figure 5:**
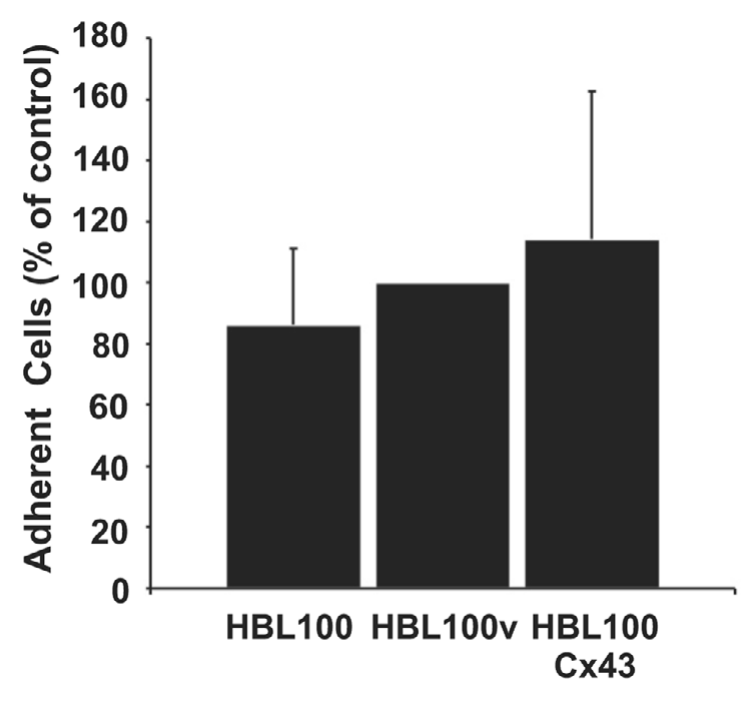
Connexin43 (Cx43) expression does not affect tumor cell adhesion to the endothelium. HBL100, HBL100v and Cx43 expressing HBL100 cells (HBL100Cx43) were labeled with dioctadecyl-3, 3,3', 3'-tetramethylindocarbocyanine percholate (DiI), added for 1 h to endothelial monolayers and co-cultures were washed to remove non-adherent cells. Fluorescence intensity measurements revealed no significant difference in the number of tumor cells attached to the endothelium regardless of cell type. Data are expressed as mean percentage ± SEM of three independent experiments with eight replicates.

To determine whether the increase in diapedesis efficiency was due to increased adhesive properties of Cx43 expressing tumor cells, we co-cultured HBL100, HBL100v and HBL100Cx43 cells on endothelial cells and compared the number of DiI labeled tumor cells adherent to HMVEC monolayers using fluorescence intensity measurements. No significant difference in the ability of tumor cells to adhere to the endothelium was observed between HBL100 breast tumor cells expressing Cx43 and tumor cells lacking Cx43 expression (Fig. 5), suggesting that the increased efficiency of diapedesis was not due to increased cellular adhesion.

**Figure 6:**
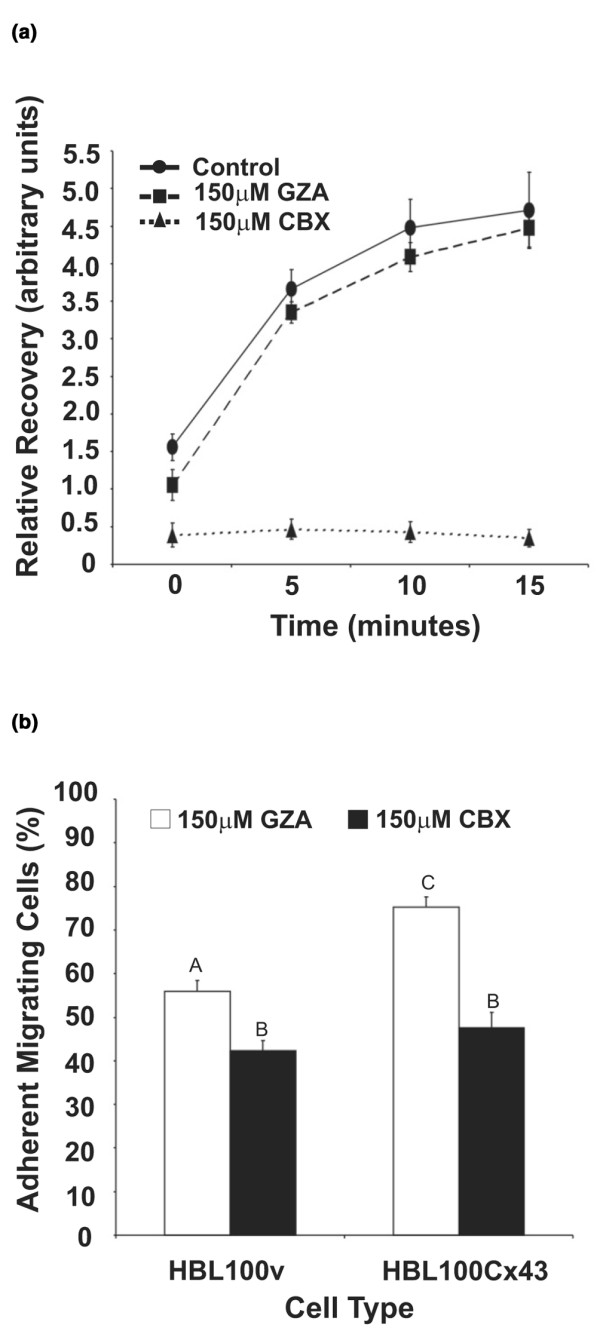
Carbenoxolone (CBX) blocks gap junctional intercellular communication (GJIC) in human microvascular endothelial cell (HMVEC) monolayers and decreases tumor cell diapedesis. **(a) **To determine the extent by which GJIC in HMVEC was blocked by CBX, monolayers were pre-treated with 150 μM CBX or the inactive analog glycyrrhizic acid (GZA) and analyzed by fluorescence recovery after photo-bleaching (FRAP). FRAP analysis revealed that fluorescence recovery by homocellular gap junctional coupling was blocked by 150 μM CBX (triangles), while recovery of fluorescence was observed in untreated (circles) or GZA treated monolayers (rectangles). Data represent three independent experiments. **(b) **To determine the effect of blocking gap junctional coupling on diapedesis, HMVEC monolayers were pre-treated with 150 μM CBX, or the inactive analog GZA and HBL100v cells or cells expressing functional Cx43 (HBL100Cx43) were co-cultured for 7 h with HMVEC monolayers, fixed and labeled for F-actin. Tumor cells were scored according to the criteria specified in Fig 1. In comparison to the inactive analog (GZA), treatment with CBX resulted in a significant decrease in the number of migrating HBL100v and HBL100Cx43 cells. Data are expressed as the mean percentage of three independent experiments ± SEM performed in duplicate or triplicate. Identical letters over bars indicate no statistical significance while different letters signify statistical significance (p < 0.01).

To determine whether GJIC was required to increase HBL100 tumor cell diapedesis, we chemically blocked gap junctional coupling using CBX. HMVEC monolayers were preloaded with calcein dye and fluorescence recovery after photobleaching (FRAP) analysis was first used to determine whether CBX effectively blocked gap junctions in endothelial cells. Individual cells within the calcein-AM loaded monolayer were bleached and recovery of fluorescence was quantified. Our results demonstrated that, in the presence of 150 μM CBX, fluorescence recovery in the bleached cell was prevented (Fig. 6a, triangles), indicating lack of homocellular gap junctional coupling. In GZA treated monolayers (negative control), however, recovery of fluorescence occurred at levels similar to those in untreated cells (Fig. 6a, rectangles and circles, respectively), indicating functional homocellular gap junctional coupling between adjacent endothelial cells.

To determine whether CBX would impair the Cx43-induced increase in diapedesis of HBL100Cx43 cells, HMVEC monolayers were pretreated with 150 μM CBX, or 150 μM GZA prior to adding tumor cells (Fig. 6b). In the presence of GZA HBL100Cx43 cells were more efficient at diapedesis compared to HBL100v control cells. A significant decrease in diapedesis efficiency in the presence of the gap junctional blocker CBX, however, was observed for both HBL100v and HBL100Cx43 cells. This finding suggests that gap junctional coupling between endothelial cells partially regulates diapedesis efficiency of tumor cells regardless of their Cx43 expression.

**Figure 7:**
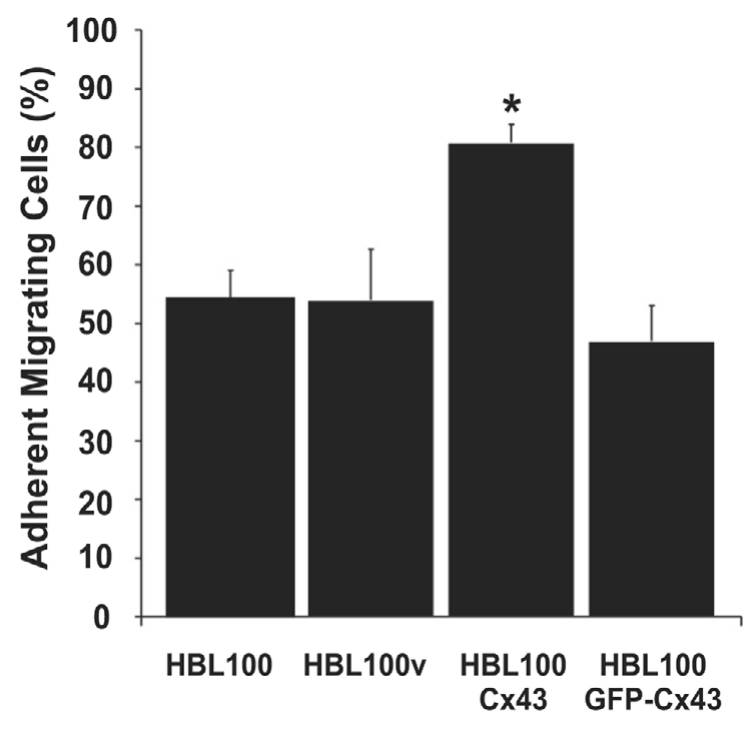
Non-functional Connexin43 (Cx43) does not enhance tumor cell diapedesis. HBL100 cells were transfected with Cx43 to which green fluorescent protein (GFP) was fused at the amino terminus (GFP-Cx43) to make it non-functional. Diapedesis of HBL100v cells and cells expressing functional (HBL100Cx43) or non-functional (HBL100GFP-Cx43) Cx43 was scored following co-culture with human microvascular endothelial cell (HMVEC) monolayers for 7 h. Analysis revealed that HBL100 cells transfected with GFP-Cx43 had an efficiency of diapedesis similar to HBL100v control cells in contrast to HBL100Cx43 cells, which showed significantly greater numbers of migrating cells. Data are expressed as the mean percentage ± SEM of five independent experiments in duplicate or triplicate (* p < 0.05).

To determine if GJIC was necessary for Cx43-linked increase in diapedesis and, more specifically, if heterocellular GJIC was necessary between tumor and endothelial cells, we compared diapedesis of HBL100 cells that expressed either the functional or non-functional Cx43 (GFP-Cx43). Previous studies showed that GFP fused to the amino terminus of Cx43 (GFP-Cx43) results in a chimeric connexin that can traffic and form gap junction-like structures at cell-cell contacts, but these structures are not functional [33] and also functional hemichannels do not form [43]. HBL100, HBL100v, HBL100Cx43, or HBL100GFP-Cx43 cells were co-cultured with HMVEC for 7 h and scored according to the described criteria for stages of migration (Fig. 1). After 7 h of co-culture the percentage of migrating HBL100GFP-Cx43 cells was similar to that of migrating HBL100 and HBL100v cells, whereas diapedesis efficiency of HBL100Cx43 cells was more than 50% higher (Fig. 7). This result suggests that the presence of Cx43 protein in the tumor cell alone is not responsible for the observed increase in diapedesis, but that heterocellular GJIC between tumor cells and endothelial cells is required to augment the efficiency of diapedesis of HBL100 cells.

## Discussion

Previous work by Kramer and Nicolson [44] evaluated melanoma cell diapedesis through endothelial monolayers by time-lapse, phase-contrast, scanning and transmission electron microscopy. In our study we used an *in vitro *cell culture model to evaluate the role of Cx43 expression in diapedesis of breast tumor cells through the endothelium by confocal microscopy. Our *in vitro *cell culture model enables us to dissect various stages of diapedesis and to examine the molecules or mechanisms involved in each of these stages [9,35,37,39-41]. Detailed morphological analysis by confocal microscopy allowed us to classify HBL100 breast tumor cell diapedesis into three stages (Fig. 1). First HBL100 breast cancer cells adhered to the endothelium but maintained a round morphology while often extending finger-like processes. This is similar to the behavior of PC3 prostate tumor cells [11] but differs from our previous findings with WM239 melanoma cells, which tended to spread along the endothelial cell surface before diapedesis and extended clusters of blebs at the heterotypic contact site between tumor cells and endothelial cells [9]. It is, therefore, likely that the adhesive interactions between tumor cells and the mechanisms of communication between endothelial cells and tumor cells are cell type specific and may regulate important aspects of diapedesis. During diapedesis, HBL100 cells, like melanoma cells, sent out projections through the endothelium, resulting in a localized retraction of the endothelial cells [9]. Compared to melanoma cells, the width of the opening through which HBL100 cells migrate appeared to be smaller and is lined by F-actin. HBL100 cells that came into contact with the underlying basement membrane spread and flattened out, forming stress fibers on the extracellular matrix, while the endothelium reformed cell-cell junctions over the top of the tumor cell. This stage of diapedesis is very similar in WM239 melanoma cells and PC3 prostate tumor cells [9,11]. Exogenous expression of Cx43 in HBL100 cells did not appear to have an effect on their adhesive properties to endothelial surfaces nor their morphology at any of the stages of diapedesis.

Interestingly, glioma cells engineered to express Cx43 formed bigger homocellular aggregates compared to control cells [45], suggesting that increased aggregation may be due to increased adhesive properties of the cells as a result of Cx43 expression.

In our study we demonstrated that HBL100 breast cancer cells engineered to express Cx43 were able to localize Cx43 protein to cell-cell contacts during diapedesis, forming communication competent gap junctions with endothelial cells (Fig. 3b,g–j). Previous studies by El-Sabban *et al*. [15] using melanoma cells expressing Cx43 support these findings, reporting that highly metastatic melanoma cells with higher levels of Cx43 mRNA coupled more efficiently to endothelial cells than low metastatic tumor cells with low amounts of Cx43 mRNA [15]. Although these and other reports demonstrated gap junctional coupling between tumor cells and endothelial cells [15-17] it was unclear if GJIC was required for increased tumor cell diapedesis. Whereas previous reports have shown Cx43 localized at contact sites between tumor cells and endothelial cells [16], our study demonstrated for the first time that Cx43 protein can be localized at the cell-cell interface between endothelial cells and tumor cells during the process of diapedesis. This localization of Cx43 to tumor/endothelial cell boundaries and the establishment of functional heterocellular coupling indicated that GJIC facilitates diapedesis. This finding is surprising because previous work has demonstrated a downregulation of connexin expression and gap junctional communication in primary tumor [29,46-49]. In addition, other studies have demonstrated reintroduction of connexins in primary tumor cells acts as a tumor suppressor in several cancers [34,50-52]. It is conceivable that tumor cells with low levels of connexins may encounter factors within the circulation such as growth factors or cytokines that could restore connexin expression previously lost in the primary tumor. For example it has been shown that vascular endothelial growth factor (VEGF), IL-1β and IL-6 are upregulated in serum of cancer patients [53,54] and that VEGF and IL-1β can increase Cx43 expression [55,56]. Increasing connexin expression in poorly invasive tumor cells, therefore, may increase their ability to migrate through the endothelium, allowing them to invade other tissues where they may develop into more aggressive tumors.

Our study shows that HBL100 breast tumor cells expressing Cx43 migrated more efficiently through the endothelium than their counterparts lacking Cx43 expression. This finding suggests that gap junctions and/or Cx43 expression in these cells may affect the motile behavior of cells by changing the kinetics of diapedesis of individual cells. The fact that Cx43 expression can influence the motile behavior of cells has been previously demonstrated [57]. For example, HeLa cells transfected with Cx43 formed functional gap junctions with embryonic chicken heart cells, and were more invasive into the ventricle of the heart compared to their wild-type counterparts, supporting the hypothesis that Cx43 expressing cells can be more invasive [57]. In addition, Cx26 expressing BL6 mouse melanoma cells had increased metastatic properties *in vivo *[17].

Cellular motility may also be affected by changes in cadherin expression. Down regulation of E-cadherin expression or function correlates with tumor development and malignancy [58,59], while N-cadherin expression appears to enhance cell motility [60,61]. Interestingly, both Cx43 and N-cadherin appear to regulate mouse neural crest cell motility, perhaps by engaging p120ctn signaling [62]. At present, a functional relationship between cadherin expression and Cx43 expression in breast tumor cell motility is largely unknown and the role of ZO-1 or p120ctn, which interact with both cadherins and connexins, in this process needs to be examined.

It was important to distinguish whether the observed increase in diapedesis efficiency was due to enhanced expression of Cx43 in the tumor cell or due to the exchange of signals between tumor and endothelial cells via functional gap junctions. Two approaches were used to examine this issue. First, we blocked homocellular and heterocellular GJIC. In the presence of the gap junctional blocker, diapedesis efficiencies were significantly reduced in both the control HBL100v cells and the Cx43 expressing HBL100 cells (HBL100Cx43) (Fig. 6b), suggesting gap junctional coupling between the adjacent endothelial cells is important in regulating tumor cell diapedesis efficiency regardless of their connexin content. Disruption of homocellular endothelial GJIC may compromise the normal function of the endothelium required for blood borne cells to cross this barrier. Our findings therefore support the hypothesis that the endothelium plays an active role in the process of diapedesis in line with previous work demonstrating that during diapedesis endothelial cells require N-cadherin to reform the junctions above melanoma cells [9]. In the second approach, heterocellular coupling between tumor cells and endothelial cells was specifically disrupted in HBL100 breast tumor cells by expressing a non-functional Cx43 in which GFP was tagged to the amino terminus of the Cx43 polypeptide. This chimeric connexin is capable of forming gap junction-like structures at the cell surface but incapable of forming functional gap junction channels or functional hemichannels as revealed by dye [33] and electrical [43] coupling assays. Our findings reveal that the diapedesis efficiency of tumor cells expressing the non-functional Cx43 was similar to that of wild-type and empty vector control cells (Fig. 7), suggesting that heterocellular GJIC between tumor and endothelial cells is necessary to enhance diapedesis. It is possible that fusing GFP to the amino terminus of Cx43 may be blocking Cx43 binding proteins that could be required for efficient tumor cell diapedesis, although, to date, no amino-terminal Cx43 binding proteins have been identified. Together with our finding that CBX decreases the efficiency of tumor cell diapedesis, our results support the hypothesis that GJIC facilitates tumor cell diapedesis.

Several possible scenarios could explain how GJIC might modulate tumor cell diapedesis. Tumor cells might send signals to the endothelium, resulting in the release of endothelial proteolytic enzymes, facilitating endothelial cell retraction. A recent study by Bazarbachi *et al. *[63] demonstrated that neoplastic lymphocytes triggered increased matrix metalloproteinase activity in endothelial cells. This activity was reduced in the presence of the gap junctional blocker 18α glycyrrhetinic acid [63]. Tumor cells may also send signals, such as inositol triphosphate (IP_3_), through gap junctions that directly trigger the weakening of inter-endothelial junctions. IP_3 _can pass between adjacent cells through gap junctions [64] and regulate intracellular calcium levels [65]. Interestingly, the depletion of calcium stores in the endothelium inhibited extravasation of MCF-7 tumor cells [66] and elevated levels of intracellular calcium resulted in the transient phosphorylation of myosin light chain [67,68], which is known to increase endothelial permeability [69]. Endothelial cells may also send signals through gap junctions to tumor cells, resulting in the upregulation of proteins involved in cellular motility (such as Rho GTPases, protein kinase C, and phosphoinositide 3 kinase).

Although it has been suggested that extravasation may not be a limiting factor for metastasis [70-72], metastatic ability appears to be correlated with the capability of tumor cells to communicate with endothelial cells as previously demonstrated in metastatic B16-F10 melanoma cells [15-17]. Transient re-expression of connexins in tumor cells, and the exchange of molecules between tumor cells and endothelial cells through gap junctions, might also affect tumor cells in their behavior following extravasation when these cells interact with the extracellular matrix and form secondary tumors. For example, melanoma cells invading into the dermis (i.e. towards the endothelium) have an increase in Cx26 expression compared to those located in the basal layer of the epidermis [17]. These invading melanoma cells showed stronger heterocellular coupling to the endothelium than homocellular coupling with each other (*in vitro*). Furthermore, squamous cell carcinomas exhibited reduced levels of Cx26 and Cx43 expression at early stages of mouse skin carcinogenesis and sites of invasion, although Cx26 expression was partially restored in these cells at metastatic sites within the lymph nodes [73].

## Conclusion

Our work supports the hypothesis that heterocellular gap junctional coupling between tumor cells and endothelium may regulate the extravasation of at least some poorly invasive tumor cells such as HBL100 breast tumor cells, and that homocellular GJIC amongst endothelial cells may play an active role in this process. These studies would suggest that even though extravasation may not necessarily be a rate limiting step in metastasis, there might be therapeutic advantages to designing drugs or reagents that inhibit vascular homocellular and heterocellular GJIC as a means of reducing metastatic spread of tumor cells.

## Abbreviations

BSA = bovine serum albumin; CBX = carbenoxolone; Cx = connexin; DiI = dioctadecyl-3, 3,3', 3'-tetramethylindocarbocyanine percholate; D-MEM = Dulbecco's modified Eagles medium; EGM = endothelial growth media; FBS = fetal bovine serum; FRAP = fluorescence recovery after photobleaching; GFP = green fluorescent protein; GJIC = gap junctional intercellular communication; GZA = glycyrrhizic acid; HBL100Cx43 = HBL100 cells expressing Cx43; HBSS = Hanks balances salt solution; HMVEC = human microvascular endothelial cell; IL = interleukin; IP_3 _= inositol triphosphate; LSCM = laser scanning confocal microscopy; PBS = phosphate buffered saline; VE = vascular endothelial; VEGF = vascular endothelial growth factor.

## Competing interests

The author(s) declare that they have no competing interests.

## Authors' contributions

MAP participated in the design of the study, carried out the imaging, immunocytochemistry, immunoblotting, and migration assays in this study, and drafted the manuscript. QS carried out the molecular biology of the study. DWL participated in the design of the study. MS conceived the study, and participated in its design and coordination and revised the manuscript. All authors read and approved the final manuscript.
